# Special Issue: NMR-Based Metabolomics

**DOI:** 10.3390/molecules26113283

**Published:** 2021-05-29

**Authors:** Miriam Pérez-Trujillo, Toby J. Athersuch

**Affiliations:** 1 Nuclear Magnetic Resonance Facility, Universitat Autònoma de Barcelona, 08193 Barcelona, Spain; 2 Department of Metabolism, Digestion and Reproduction, Faculty of Medicine, Imperial College London, London SW7 2AZ, UK

Nuclear magnetic resonance (NMR) spectroscopy remains one of the core analytical platforms for metabolomics, providing complementary chemical information to others, such as mass spectrometry, and offering particular advantages in some areas of research on account of its inherent robustness, reproducibility, and phenomenal dynamic range. While routine experimental protocols for profiling and related statistical analysis pipelines have been established, they often present considerable challenges to the analyst, including spectral overlap, accurate and precise quantification, and chemical shift variation. Consequently, there is still much activity across all areas of NMR spectroscopic analysis in relation to metabolomics. Furthermore, there remain many biological systems and sample types that have not been extensively explored using NMR spectroscopy-based metabolomics.

In this Special Issue, several advances in methodology, and new applications in the field of NMR-based metabolomics, have been presented. In addition, the SI includes authoritative review articles focused on the state-of-the-art of quantitative NMR spectroscopy in biomedical metabolomics applications, and novel applications in the agri-food sector.

The use of new NMR spectroscopic pure shift methods in NMR spectroscopy-based metabolomics is evaluated by Stark et al. [1] in *Hypericum* plant extracts; pure shift methods improve spectral resolution in ^1^H-NMR by suppressing homonuclear coupling, turning multiplets into singlets. They test the PSYCHE (*p*ure *s*hift *y*ielded by *ch*irp *e*xcitation) and the Zangger–Sterk pulse sequences with conventional ^1^H-NMR spectroscopic sequences used routinely in profiling and suggest the implementation of such methods in the follow-up experiments of the metabolomics workflow, as a complementary data source that can aid data interpretation. In their SI paper, Mehendale et al. [2] describe the development of a novel NMR-compatible platform for the automated real-time monitoring of biochemical reactions using a flow shuttling configuration. This system offers high-resolution analysis, automation, minimal user intervention, and, therefore, time-efficient measurement per sample. The platform described suits bioprocessing applications, in which conversion to a value-added product is the objective, since the intermediate and final product yields can be monitored in real time.

Gargallo-Garriga et al. [3] conducted metabolic profiling using ^31^P NMR spectroscopic methods in a study of phosphorus utilization in the trees of a French Guiana rainforest; the productivity and diversity of tropical lowland moist forests are often limited by the availability and functional allocation of phosphorus, and thus ^31^P NMR offers a direct window on the relevant aspects of plant biochemistry. The authors conclude that phosphorus use is species-specific and that species diversity relates to both species phosphorus use and concentrations of phosphorus-containing compounds.

In their contribution, Kim et al. [4] investigate meat-aging processes, specifically the characteristic metabolic changes of the crust from dry-aged beef using 2D quantitative NMR-based metabolomics. Multivariate analysis of NMR spectra from various examples of aged meats allows the method, degree, and doneness of the aging process to be effectively differentiated and the key discriminatory metabolites to be identified. Special attention is given to the investigation of methylmalonic aciduria (MMA) by Nicolescu et al. [5]. They report on monitoring MMA-specific and non-specific metabolites via NMR-based metabolomics and present an extended dataset of targeted metabolites.

The current status and applications of quantitative NMR-based biomedical metabolomics is reviewed by Crook et al. [6]. The authors highlight the significant improvements in metabolite quantification that have taken place in recent years. They cover advances in the use of reference standards, pulse sequence development, physical and chemical deconvolution methods, hyperpolarization techniques, physical and chemical deconvolution methods, as well as the integration of multiple analytical platforms.

Finally, particular attention is given to applications in the agri-food sector. The discrimination of the variables influencing tomato (*Solanum lycopersicum*) using NMR-based metabolomics is reviewed by Abreu et al. [7]. The knowledge derived from the reported studies is especially helpful in improving fruit quality through cultural practices and agronomical conditions.

## References

[1] Stark P., Zab C., Porzel A., Franke K., Rizzo P., Wessjohann L.A. (2020). PSYCHE—A Valuable Experiment in Plant NMR-Metabolomics. Molecules.

[2] Mehendale N., Jenne F., Joshi C., Sharma S., Masakapalli S.K., MacKinnon N. (2020). A Nuclear Magnetic Resonance (NMR) Platform for Real-Time Metabolic Monitoring of Bioprocesses. Molecules.

[3] Gargallo-Garriga A., Sardans J., Llusià J., Peguero G., Asensio D., Ogaya R., Urbina I., Langenhove L.V., Verryckt L.T., Courtois E.A. (2020). ^31^P-NMR Metabolomics Revealed Species-Specific Use of Phosphorous in Trees of a French Guiana Rainforest. Molecules.

[4] Kim H.C., Baek K.H., Ko Y.-J., Lee H.J., Yim D.-G., Jo C. (2020). Characteristic Metabolic Changes of the Crust from Dry-Aged Beef Using 2D NMR Spectroscopy. Molecules.

[5] Nicolescu A., Blanita D., Boiciuc C., Hlistun V., Cristea M., Rotaru D., Pinzari L., Oglinda A., Stamati A., Tarcomnicu I. (2020). Monitoring Methylmalonic Aciduria by NMR Urinomics. Molecules.

[6] Crook A.A., Powers R. (2020). Quantitative NMR-Based Biomedical Metabolomics: Current Status and Applications. Molecules.

[7] Abreu A.C., Fernández I. (2020). NMR Metabolomics Applied on the Discrimination of Variables Influencing Tomato (*Solanum lycopersicum*). Molecules.

